# Reactivity of Autologous Serum IgG to Gut Microbes in Pediatric Ulcerative Colitis

**DOI:** 10.3390/ijms26178196

**Published:** 2025-08-23

**Authors:** Nafisa Tabassum, Haruyuki Nakayama-Imaohji, Emmanuel Munyeshyaka, Ayano Tada, Takeo Kondo, Sonoko Kondo, Takashi Kusaka, Tomomi Kuwahara

**Affiliations:** 1 Department of Microbiology, Faculty of Medicine, Kagawa University, 1750-1 Miki-cho, Kita-gun, Takamatsu 761-0793, Kagawa, Japan; s22d720@kagawa-u.ac.jp (N.T.); imaoji.haruyuki@kagawa-u.ac.jp (H.N.-I.); s22d730@kagawa-u.ac.jp (E.M.); tada.ayano@kagawa-u.ac.jp (A.T.); 2 Department of Pediatrics, Faculty of Medicine, Kagawa University, 1750-1 Miki-cho, Kita-gun, Takamatsu 761-0793, Kagawa, Japanijichi.sonoko@kagawa-u.ac.jp (S.K.); kusaka.takashi@kagawa-u.ac.jp (Takashi Kusaka)

**Keywords:** serum IgG, pediatric UC, gut microbes, colonoscopy, *Escherichia coli*, *Lactobacillus* spp.

## Abstract

Ulcerative colitis (UC) is caused by an excessive immune response to gut microbiota. A recent study reported that the population of IgG-coated gut microbes increases with disease severity in patients with UC, but the role of these IgG-coated microbes in UC pathology is unclear. Serum, feces and colonoscopic lavage fluids (CLFs) were collected from pediatric UC (*n* = 13) and non-inflammatory bowel disease (IBD) patients (*n* = 15). Gut microbes were isolated from feces. Serum IgG reactivity to microbial cells and CLF-derived proteins was evaluated by Western blotting. Complement activation by the bacteria–IgG complexes was also assessed. Serum IgG reactivity to gut microbial extracts was highly variable in patients with active UC and increased with mucosal inflammation. IgG reactivity and clinical condition were inversely associated depending on disease activity. Non-IBD patients showed a similar degree of serum IgG response as that seen for patients whose UC was in remission. Lactobacillaceae bound higher amounts of IgG than other gut microbes tested and absorbed IgG to other bacteria. *Lacticaseibacillus paracasei* suppressed complement activation by *Escherichia coli*—IgG immune complexes. Appropriate IgG responses to luminal microbes might play a key role in gut microbiota stability by reducing excessive mucosal inflammation.

## 1. Introduction

Ulcerative colitis (UC) is a chronic inflammatory disease of the gastrointestinal tract. The exact pathogenesis of UC remains largely unclear, but multiple factors including genetic susceptibility, gut microbial dysbiosis and living environment are associated with UC onset [1,2,3]. Much of the impact of UC is due to the complex nature of the disease pathogenesis and delayed diagnosis [4,5,6].

UC is characterized by disruption of the gut mucosal barrier, excessive immune response to gut microbes and deterioration of the gut microbiota function needed to maintain intestinal homeostasis, which together result in a negative loop of prolonged gut microbial dysbiosis [7,8,9]. In a healthy gut, luminal microbes are separated from epithelial cells by the mucous layer, which contains antimicrobial substances and immunoglobulins [10,11,12]. Gut microbiota can be divided into two populations: luminal microbes (LM) and mucosa-associated microbes (MAM) [13]. Recent studies demonstrated the marked heterogeneity of microbial communities among colonic mucosa and stool samples [14,15]. Contamination of the gut mucous layer by LM, crypt abscesses and direct contact of gut microbes with colonic epithelial cells are representative pathological changes that occur in UC [16,17]. Alterations in MAM in inflammatory bowel diseases (IBDs) have been extensively studied using biopsy samples or lavage fluids collected during colonoscopy [15,18]. Operational taxonomic units (OTUs) including mucin-degrading *Fusobacterium* spp. have been shown to be enriched in biopsy samples [17,19]. Another approach to identifying the pathophysiological role of gut microbes in UC involves analysis of IgA- or IgG-coated bacteria, which are thought to elicit host immune responses [20,21]. Armstrong et al. performed a metagenomic analysis of these complexes using terminal ileum aspirates from pediatric patients with UC and found that IgG tended to preferentially bind *Burkholderia cepacia*, *Flavonifractor plautii* and *Ruminococcus* spp., which were demonstrated to have invasive potential toward human intestinal cells in vitro [2].

IgA and IgG secreted into the intestinal mucous layer can bind pathogens and impede their ability to reach gut epithelial cells. Neonatal Fc receptors (FcRns) expressed on gut epithelial cells are reported to mediate both the absorption and secretion of IgG [22]. Several studies have shown that the percentages of IgA- or IgG-coated bacteria are altered depending on the disease activity of IBD [23]. Secretory IgA (sIgA) is the most abundant immunoglobulin found in luminal fluid and IgG is the second most abundant. As such, multiple previous studies have focused on IgA-coating bacteria to identify etiologic microbes for UC [3]. However, due to its broad antigen specificity, IgA binds even to commensal bacteria, which complicates the identification of the specific microbes that induce excessive host immune responses [24]. Recent studies have reported that more IgG^+^ plasmablasts are present in the intestinal tract than IgA^+^ plasmablasts, and that the bacterial epitope recognition of IgG is higher than that of IgA, leading to a three-fold higher reactivity [25]. Together, these factors suggest that luminal IgG plays a major role in blocking the translocation of specific bacteria that have higher immunogenicity, which is consistent with the finding that the population of IgG-coated fecal bacteria increases with UC disease activity while the population of IgA-coated bacteria remains unchanged [26].

In this study, we investigated the reactivity of autologous serum IgG to gut microbes in pediatric patients with UC by analyzing fecal isolates and colonoscopic lavage fluids (CLFs) collected from ascending (A), transverse (T) and sigmoid (S) parts of the colon and rectum (R).

## 2. Results

### 2.1. Reactivity of Serum IgG to Protein Extracts from CLFs or Feces from Pediatric Patients with UC

**Figure 1:**
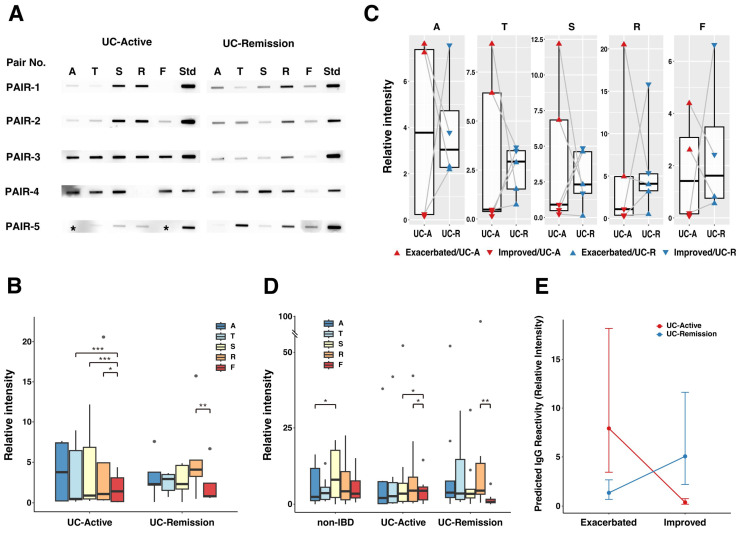
Comparison of serum IgG reactivity to gut microbial antigens. Western slot blot against microbial antigens in CLFs collected from different parts of the colon (A, ascending colon; T, transverse colon; S, sigmoid colon and R, rectum) and feces (F) was performed with purified serum IgG from pediatric UC and non-IBD patients. UC disease activity (active or remission) was assessed according to PUCAI and clinical findings. (**A**) Photographic representation of the Western slot blot for the 5-pair cohort, which includes five pediatric UC patients from whom the samples were collected during both active and remission stages. Human IgG (Normal Human IgG, Fujifilm) was used as an internal standard (shown as Std) for inter-membrane comparison. Missing samples are indicated by asterisks (*). (**B**) Comparison of serum IgG reactivity at each anatomical site in the 5-pair cohort, stratified by disease activity. Relative serum IgG reactivity (Relative intensity) to gut microbial antigens was calculated by dividing the intensity of the sample band by that of the standard (Std). For the 5-pair cohort, serum IgG reactivity is shown as boxplots for each anatomical site (A, T, S, R, F), separated by disease activity (UC-Active and UC-Remission). Asterisks indicate statistically significant differences between sites within each disease activity (GLMM with Tukey’s post hoc test; *, *p* < 0.05; **, *p* < 0.01; ***, *p* < 0.001). (**C**) Relationship between serum IgG reactivity, disease activity and clinical condition at each anatomical site. Serum IgG reactivity during the active and remission stages is shown as boxplots for each site. Individual data points have different colors and shapes according to disease activity (red: UC-Active, blue: UC-Remission) and clinical condition (▲: Exacerbated, ▼: Improved). Lines connect paired samples from the same patient. (**D**) Serum IgG response to gut microbial antigens in all subjects including non-IBD patients. Serum IgG reactivity to all samples is shown as boxplots for each anatomical site. Patients were separated into three cohorts: non-IBD, UC-Active and UC-Remission. Asterisks indicate statistically significant differences between sites within each cohort (GLMM with Tukey’s post hoc test; *, *p* < 0.05; **, *p* < 0.01). (**E**) Interaction of serum IgG reactivity with disease activity and clinical condition. The plot shows predicted values (estimated marginal means) of serum IgG reactivity calculated from a generalized linear mixed-effects model (GLMM). Points represent the estimated means, and error bars indicate 95% confidence intervals. A strong, significant interaction was observed between disease activity (UC-Active/UC-Remission) and clinical condition (Exacerbated/Improved) (*p* < 0.001).

Serum (*n* = 33), colonoscopic lavage fluids (CLFs, *n* = 145) and feces were collected from pediatric patients with UC (*n* = 13) and non-IBD patients (*n* = 15) after obtaining informed consent from their guardians (Table 1). For five patients with UC, serum and CLFs were collected at different stages of disease activity (Tables S1 and S2). We compared the reactivity of serum IgG to mucosa-associated microbes (MAM) in CLFs or microbes in feces from pediatric patients with active UC and/or whose disease was in remission. Extracts from CLFs and feces were first adjusted to the lowest concentration present among the samples (780 μg/mL). Western slot blot analysis was then conducted against these extracts with serum IgG. High reactivity of serum IgG to MAM- and luminal microbe (LM)-derived extracts was seen in samples from both patients with active UC and from those whose UC was in remission (Figure 1A).

The reactivity of serum IgG to CLF-derived extracts was highly variable in patients with active UC, but in the five-pair cohort (Table S2), no overall significant difference was seen in IgG reactivity between the active and remission stages (GLMM, *p* = 0.181) (Figure 1B). However, when analyzing site-specific differences stratified by disease activity, a distinct local pattern emerged, in which IgG reactivity to feces (F) was significantly lower than that for CLFs collected from the rectum (R) during the remission stage (*p* = 0.005), and lower than that for samples collected from multiple sites in the colon (R, S, and T) in patients with active disease (Figure 1B). A simple paired *t*-test, which was performed for every site individually, showed no significant differences between the disease activities (Figure 1C).

In an evaluation of the association between IgG reactivity and clinical condition (exacerbation/improvement) (Figure 1C,E, Table 2 and Table S2), a significant interaction between disease activity and clinical condition (coefficient β = 4.36, 95% CI [2.83, 5.88], *p* < 0.001; Table 2) was found. For the active stage, clinical improvement was strongly associated with a decrease in IgG reactivity, whereas in the remission stage, IgG reactivity increased. This inverse effect is clearly visualized by the intersection of the two lines in the interaction plot (Figure 1E).

We also evaluated site-specific reactivity across all samples, including a non-IBD control group (Figure 1D and Table S1). Although we observed no significant difference in the overall IgG reactivity among the three cohorts (non-IBD, active UC, remission UC), the pattern of site-specific IgG reactivity differed. In the non-IBD group, IgG reactivity in the sigmoid colon (S) was higher than in other sites, whereas in the UC groups, the reactivity to feces (F) tended to be lower compared to that for MAMs.

### 2.2. Association of Serum IgG Response with UC Disease Severity and Mucosal Inflammation

**Figure 2:**
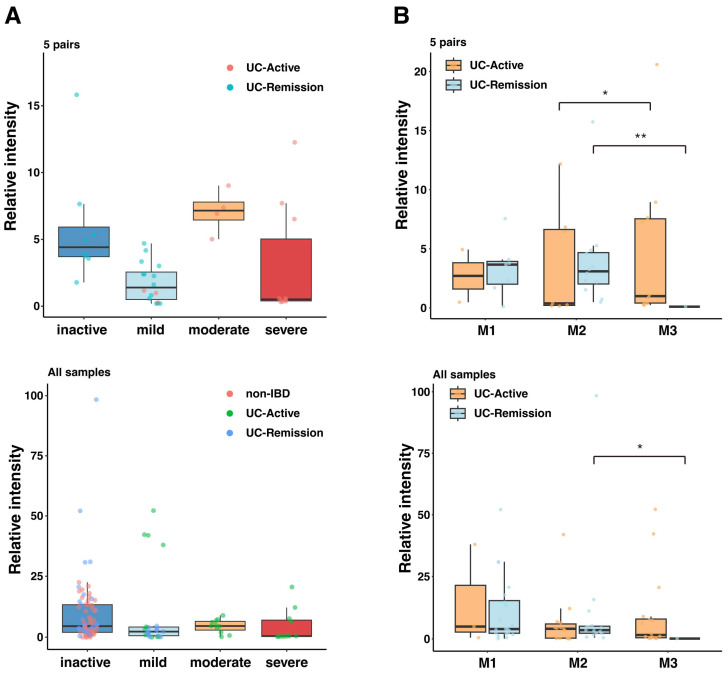
Correlation between autologous serum IgG reactivity to MAM-derived antigen with disease severity and mucosal inflammation. We examined the correlation between serum IgG reactivity to MAM-derived antigens and disease severity or endoscopic mucosal assessment (Matts score: M1, mild; M2, moderate; M3, severe). (**A**) Association between disease severity and serum IgG reactivity. Relative serum IgG reactivity is shown as boxplots across disease severity for the 5-pair UC cohort (Top panel) and all-sample cohort (Bottom panel). Disease activity of individual UC patients in the top panel is indicated by colors (red: UC-Active, blue: UC-Remission), In the bottom panel, the study groups are distinguished by colors (red: non-IBD, green: UC-Active, blue: UC-Remission). In both cohorts, no statistically significant differences were found among the groups based on disease severity (GLMM with Tukey’s post hoc test, *p* > 0.05 for all comparisons). (**B**) Association between Matts score and serum IgG reactivity. Serum IgG reactivity is shown as boxplots across the groups based on Matts score for the 5-pair cohort (top panel) and all-sample cohort (bottom panel). Disease activity of individual patients is shown with different colors (orange: UC-Active, light blue: UC-Remission). Asterisks indicate statistically significant differences between the groups (GLMM with Tukey’s post hoc test, *, *p* < 0.05; **, *p* < 0.01).

We analyzed the association between serum IgG reactivity to MAM-derived extracts and disease severity in pediatric patients with UC. Disease severity was assessed by the Pediatric Ulcerative Colitis Activity Index (PUCAI) and categorized as “inactive”, “mild”, “moderate” or “severe” (Figure 2A). All non-IBD subjects were categorized into the “inactive” group. Mucosal inflammation was assessed by endoscopy and categorized by the Matts score (M1, M2 or M3) (Figure 2B).

First, the association between IgG reactivity and PUCAI score was evaluated (Figure 2A). In analyses of both the five-pair cohort and all-sample cohort (including non-IBD controls), no statistically significant differences in IgG reactivity were found among the disease severities (GLMM with Tukey’s test, *p* > 0.05 for all comparisons). However, a sub-analysis focusing specifically on the remission stage detected a clearer trend (Figure S1). In the five-pair remission cohort, IgG reactivity tended to be lower in the mild group compared with the inactive group, although this difference did not reach statistical significance (*p* = 0.117). A statistically significant difference was observed, however, when the analysis was expanded to include all samples, wherein the mild group showed lower IgG reactivity than did the inactive group (*p* = 0.023).

Next, the association of IgG reactivity with mucosal inflammation (Matts score) was evaluated (Figure 2B). Similar to the analysis based on disease severity, no significant differences in IgG reactivity were found among the three Matts score groups in either cohort. However, a stratified analysis separating the UC active and UC remission disease stages revealed a stage-dependent relationship. In the five-pair cohort, reactivity in the M2 group was significantly lower than in the M3 group in the active stage (*p* = 0.018). In contrast, during the remission stage, this relationship was reversed, with the M2 group showing significantly higher reactivity than the M3 group (*p* = 0.012). In the analysis using all samples, no significant differences were found among Matts score groups in the active stage. However, the finding for the remission stage was consistent, with the M2 group again showing significantly higher reactivity than the M3 group (*p* = 0.044).

### 2.3. Serum IgG Reactivity to Specific Fecal Bacteria

Gut bacteria colonizing the small intestine like *Streptococcus* spp., *Enterococcus* spp., *Lactobacillus* spp. and *Veillonella* spp. were previously shown to be predominantly coated with IgG in biopsy samples [1]. Accordingly, we aimed to isolate these bacteria from the feces of pediatric patients with UC. We successfully isolated three species formerly classified into the genus *Lactobacillus* (*Lacticaseibacillus paracasei*, *Lactiplantibacillus plantarum* and *Ligilactobacillus salivarius*), *Enterococcus faecalis* and three isolates belonging to Enterobacteriaceae (*E. coli*, *Klebsiella pneumoniae* and *Proteus mirabilis*) (Table S3). We evaluated serum IgG reactivity to these isolates by Western slot blot analysis.

**Figure 3:**
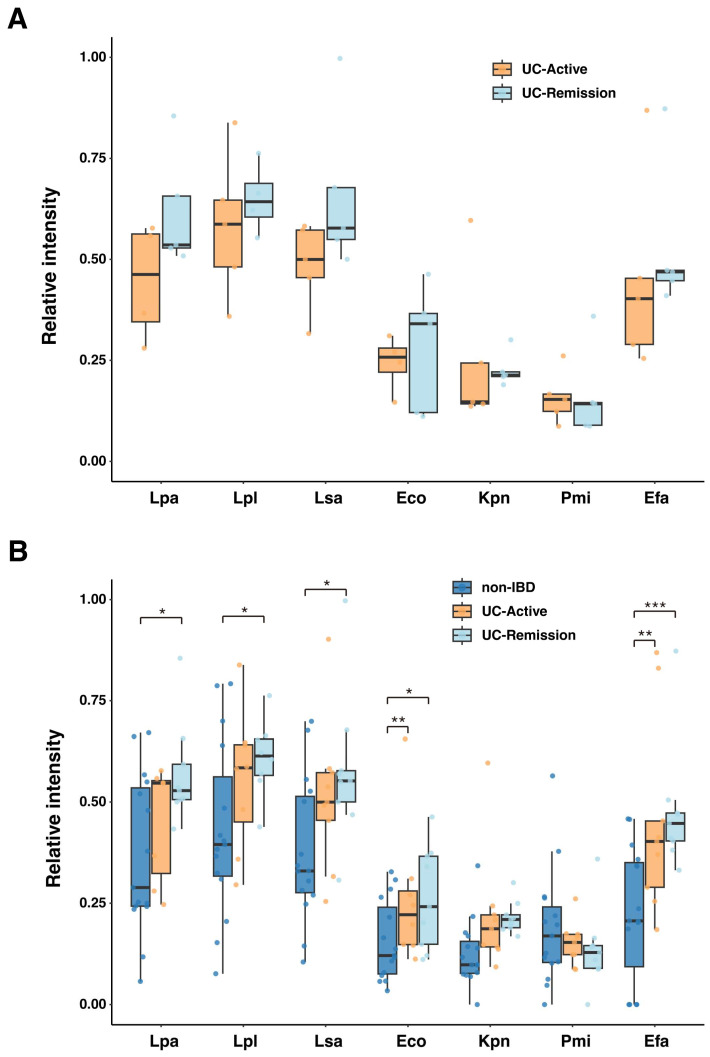
Serum IgG reactivities to selected fecal bacteria. Abbreviations: Lpa, *Lactocaseibacillus paracasei*; Lpl, *Lactiplantibacillus plantarum*; Lsa, *Ligilactobacillus salivarius*; Eco, *Escherichia coli*; Kpn, *Klebsiella pneumoniae*; Pmi, *Proteus mirabilis*; Efa, *Enterococcus faecalis*. (**A**) Comparison of serum IgG reactivity to each gut microbe by disease activity in the 5-pair cohort. For the 5-pair cohort, serum IgG reactivity is shown as boxplots for each gut microbe. Disease activities of individual patients are indicated by colors of the plots (orange: UC-Active, light blue: UC-Remission). The post hoc multiple comparisons revealed no statistically significant differences between the active and remission stages for any of the microbes (GLMM with Tukey’s post hoc test, *p* > 0.05 for all comparisons). (**B**) Comparison of serum IgG reactivity to each gut microbe across non-IBD, UC-active and UC-remission cohorts. Serum IgG reactivity from all samples is shown as boxplots for each gut microbe. Data are separated into three cohorts: non-IBD (dark blue), UC-active (orange) and UC-remission (light blue). Asterisks indicate statistically significant differences between groups following Tukey’s multiple comparison test (GLMM; *, *p* < 0.05; **, *p* < 0.01; ***, *p* < 0.001).

The fecal bacteria described above were fixed with 4% paraformaldehyde (PFA) and blotted onto PVDF (polyvinylidene difluoride) membranes using a slot blotter. We examined the serum IgG reactivity to these bacteria in the five-pair cohort of UC patients from whom serum was collected during both the active and remission stages. High reactivity was observed against *L. paracasei* (Lpa), *L. plantarum* (Lpl), *L. salivarius* (Lsa), *E. coli* (Eco) and *E. faecalis* (Efa), whereas reactivity to *K. pneumoniae* (Kpn) or *P. mirabilis* (Pmi) was low (Figure 3A). Statistical analysis using GLMM detected no significant differences in IgG reactivity between active and remission stages for any of the microbes. Next, we compared IgG reactivity between three cohorts that included non-IBD controls (Figure 3B). Consistent with the five-pair cohort analysis, we saw no significant differences between the UC active and UC remission groups for any of the microbes. However, IgG reactivity to many of the microbes in the UC groups was significantly higher than that for the non-IBD group. Specifically, for Eco and Efa, both UC active and UC remission groups showed significantly higher reactivity than the non-IBD group (*p* < 0.05). For Lpa, Lpl and Lsa, the remission UC group in particular showed significantly higher reactivity than the non-IBD controls (*p* = 0.035, *p* = 0.031 and *p* = 0.043, respectively). No significant differences were observed among the three groups for Kpn and Pmi. These results suggest that a breakdown of immune tolerance to commensal gut microbiota is a persistent immunological feature of pediatric UC, regardless of disease activity.

### 2.4. Serum IgG Reactivity to Selected Fecal Microbes After Absorption by L. paracasei or L. plantarum

Due to the high reactivity of serum IgG to *L. paracasei* and *L. plantarum*, we hypothesized that these bacteria could cross-react with a broad range of IgG, thereby potentially suppressing subsequent inflammatory immune complexes. To test this, we compared the reactivity of serum IgG from a UC patient in both active (UCA36) and remission (UCR25) stages to various fecal bacteria before and after absorption with *L. paracasei* or *L. plantarum*.

**Figure 4:**
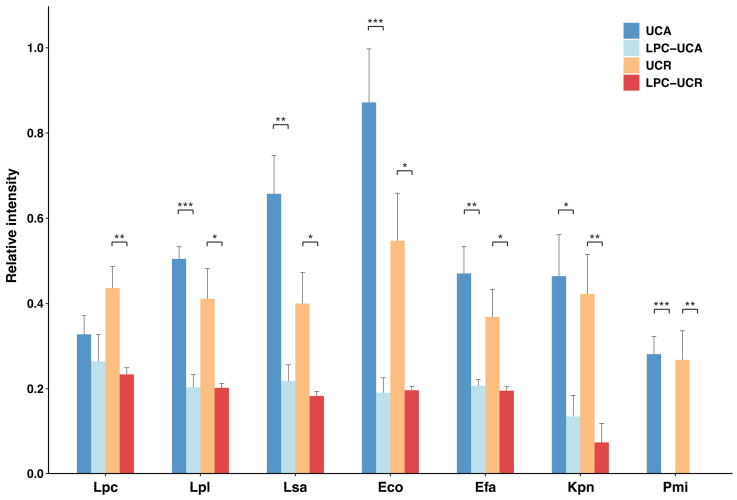
Reactivity of serum IgG to selected fecal bacteria after absorption by *L. paracasei*. Serum IgG from a pediatric patient with UC in the active stage and remission stage (UCA36 and UCR25, respectively) was pre-mixed with PFA-fixed *L. paracasei* OY7 to inhibit IgG binding to these bacteria. Reactivity of the unabsorbed (Dark blue bars: UCA, active UC; Orange bars: UCR, remission UC) and absorbed sera (Light blue bars: LPC-UCA, IgG serum from patient with active disease pre-absorbed to LPC; Red bars: LPC-UCR, IgG serum from patient in remission pre-absorbed to LPC) against protein extracts from the indicated bacterial isolates was then compared in a Western slot blot analysis. Error bars represent the standard error of the mean (SEM) from three independent experiments. Asterisks indicate a statistically significant difference between the unabsorbed and absorbed groups for each condition (two-sample *t*-test; *, *p* < 0.05; **, *p* < 0.01; ***, *p* < 0.001).

IgG absorption by *L. paracasei* OY7 altered subsequent IgG binding to the selected bacteria (Figure 4). In the remission serum (UCR25), pre-absorption with *L. paracasei* significantly reduced IgG reactivity against all tested bacteria. For active serum (UCA36), we observed a similar significant reduction in the IgG reactivity for nearly all bacterial strains. Notably, reactivity against *L. paracasei* itself was not significantly reduced for IgG from active UC serum (*p* = 0.43). On the other hand, the reactivity of absorbed serum IgG from the UC remission stage (UCR) to *L. paracasei* was significantly decreased, indicating that the humoral immune response to *L. paracasei* is lower in the UC active phase (UCA) than in UC remission (UCR).

In another absorption assay using *L. plantarum* OY1, broader cross-reactivity was observed in the UC active phase (Figure S2). For active serum (UCA36), absorption by *L. plantarum* significantly reduced subsequent IgG reactivity against a wide range of bacteria, including Lpl, Lsa, Eco, Efa, Kpn and Pmi. In contrast, for remission serum (UCR25), the degree of the reduction was reduced, with a significant reduction in IgG reactivity seen only for Kpn and Pmi. The reactivity of serum IgG to *L. paracasei* was not changed by IgG absorption to *L. plantarum* using both sera from UC active and UC remission.

### 2.5. Altered Serum IgG Reactivity to Gut Microbes After Absorption by E. coli

**Figure 5:**
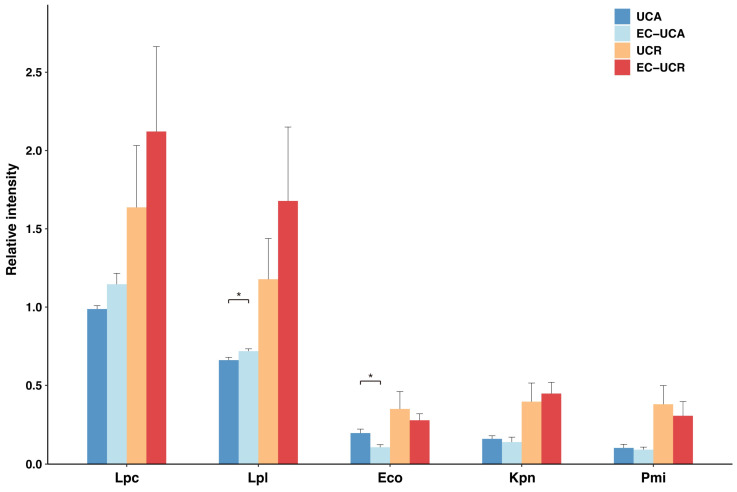
Serum IgG reactivity to selected fecal bacteria after absorption by *E. coli*. Serum IgG from a pediatric UC patient (active stage: UCA36; remission stage: UCR25) was pre-mixed with PFA-fixed *E. coli* TF10 to inhibit IgG binding to this bacterium. The reactivity of the unabsorbed and absorbed sera against the indicated bacterial isolates was then compared in Western slot blots. The color coding of the bars is the same as that in Figure 4. Error bars represent the standard error of the mean (SEM) from three independent experiments, and asterisks indicate a statistically significant difference between the unabsorbed and absorbed groups for each condition (two-sample *t*-test; *, *p* < 0.05).

We next examined the effect of *E. coli* absorption on serum IgG reactivity to selected fecal bacteria (Figure 5). For active-stage serum (UCA36), absorption by *E. coli* TF10 significantly decreased IgG reactivity against *E. coli* itself (*p* < 0.05), and, paradoxically, caused a significant increase in reactivity against *L. plantarum* (Lpl) (*p* < 0.05). For remission-stage serum (UCR25), no statistically significant changes in IgG reactivity to any of the tested bacteria were observed. However, the reactivities to *L. paracasei* and *L. plantarum* tended to be higher after absorption compared to unabsorbed serum IgG. This result could be due to the relative enrichment of IgG specific to *L. paracasei* and *L. plantarum*, which would still recognize specific epitopes on these bacteria and would not be affected by pre-absorption to *E. coli*.

### 2.6. Complement Activation by Immune Complexes Composed of UC-Derived Serum IgG and L. paracasei and/or E. coli

**Figure 6:**
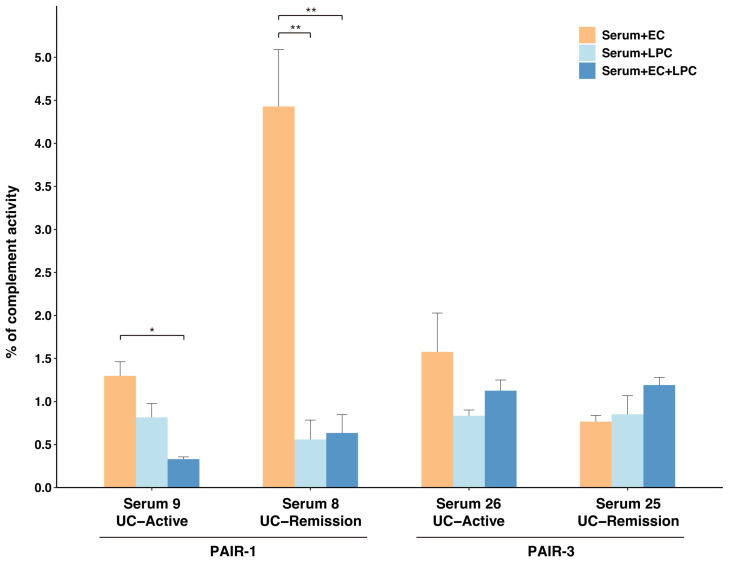
Classical complement pathway activation with immune complexes composed of serum IgG and *E. coli and/or L. paracasei*. The ability of paired sera samples collected from two pediatric patients with UC (PAIR-1 and PAIR-3) during active and remission stages to activate the classical complement pathway was compared after incubation with *E. coli* (EC; orange bars), *L. paracasei* (LPC; light blue bars), or both (dark blue bars). Data are expressed as the mean ± standard error of the mean (SEM) from three independent experiments. Within each serum sample, statistical comparisons among the three conditions were performed using a one-way ANOVA followed by Tukey’s post hoc test. Asterisks indicate statistically significant differences (*, *p* < 0.05; **, *p* < 0.01).

*L. paracasei*-absorbed IgG reduced reactivity to *E. coli* as well as to all other fecal bacteria tested in this study. Based on this result, we hypothesized that *L. paracasei* suppresses the formation of immune complexes that evoke inflammatory responses like the activation of the complement pathway or macrophages. We evaluated the capability of immune complexes between *L. paracasei* or *E. coli* and the corresponding serum IgG to activate the classical complement pathway. We selected two UC patients (PAIR-1 and PAIR-3 in Table S1) for whom both bacteria and serum samples were available at different clinical stages of disease (active or remission) (Tables S1 and S3). A high level of complement activation induced by *E. coli* and remission-stage serum (Serum 8) was significantly suppressed by co-incubation with *L. paracasei* (*p* = 0.005; Figure 6). A similar significant suppressive effect was also confirmed with active-stage serum (Serum 9) (*p* = 0.013). The other paired samples (PAIR-3) showed similar trends, but the addition of *E. coli* and IgG induced only weak complement activation.

## 3. Discussion

In this study, we examined the humoral immune response to gut microbiota residing in proximity of the epithelial layer (MAM) and luminal content (LM) in pediatric patients with UC. Compared to adults, pediatric patients with UC manifest more severe symptoms, and pancolitis is a common disease type [27]. Since the degree of mucosal inflammation defined based on endoscopic evaluation differs, as do different sites in the colon even in pancolitis cases, examining the immune response to MAMs according to mucosal inflammation is valuable. An analysis of all samples available for the study cohort showed no significant difference in IgG reactivity among the three groups (non-IBD, UC active and UC remission) (Figure 1D). In samples from patients with UC, the IgG reactivity to LM was characteristically lower than for MAM, indicating the difference in microbial compositions between MAM and LM in patients with UC. We obtained notable findings in an analysis of five patients with UC from whom samples were collected during both active disease and remission stages. First, IgG reactivities to MAM were more variable during active disease than during remission (Figure 1C,D). Second, the disease trend (exacerbation/improvement) and IgG reactivity to gut microbes showed a significant inverse interaction between active disease and remission (Figure 1E). In the active stage, a decrease in IgG reactivity to gut microbes was strongly associated with improvement in disease symptoms, suggesting that IgG reactivity may reflect the degree of inflammation in this context. In contrast, during disease remission, improvement in symptoms was associated with an increase in IgG reactivity. This inverse association suggests that the IgG response is not merely a marker of inflammation, but instead may play a different, regulatory role during remission, such as in the maintenance of mucosal homeostasis or immune surveillance. Supporting this possibility is our finding that IgG reactivity to autologous gut microbes in samples from non-IBD patients were equivalent to those seen for UC patients. Thus, IgG reactivity represents a complex immune response that differs based on the local state of the gut mucosa and qualitatively changes its functional role depending on the degree of mucosal inflammation.

IgA and IgG form a dominant class of immunoglobulins found in human luminal fluids. The detailed mechanisms by which serum IgG is secreted into mucosal surfaces are not clear. Yoshida et al. showed that serum IgG is secreted by the neonatal Fc receptor for IgG (FcRn), even in adults [22,28]. Qian et al. reviewed the role of IgG secretion into the mucous layer via FcRn in protection from infection [29]. Bleeding from inflamed mucosa in IBD is another way by which IgG could be translocated from serum to gut mucosa [1,30] and could also allow leakage of other immune components like neutrophils and complement into luminal sites. Our results of this study showed that IgG responses directed toward autologous gut microbes such as *L. paracasei*, *L. plantarum*, *E. faecalis* and Enterobacteriaceae (except for *P. mirabilis*) were significantly higher in pediatric patients with UC compared to non-IBD controls (Figure 3B), indicating a heightened humoral immune response to specific gut microbes in pediatric UC. It is particularly noteworthy that this elevated IgG response was observed both during the active stage of disease and during the remission stage. This result strongly suggests that the breakdown of immune tolerance to commensal gut microbiota is not a transient reflection of an inflammatory state, but is a stable and fundamental immunological feature of pediatric UC that persists regardless of disease activity. In other words, the immune system could remain “sensitized” to specific gut bacteria even after clinical remission is achieved and symptoms have subsided.

Adherent and invasive *E. coli* are well known as an etiologic microbe for IBD [31]. The immune response to flagella tip protein (H) in this type of *E. coli* is closely related to mucosal inflammation in IBD [32,33]. In this study, we also observed high serum IgG reactivity to Enterobacteriaceae in addition to IgG reactivity to *Lactobacillus* spp. and closely related species, which is consistent with findings by Bourgonje et al. [1]. Meanwhile, Wang et al. reported increased proportions of *Bifidobacterium* spp. and *Lactobacillus* spp. [34] in the gut mucosal layer, indicating roles for these gut microbes in IBD, even though they are generally recognized as probiotic bacteria. Notably, we showed that serum IgG reactivity to the probiotic species *L. paracasei* and *L. plantarum* was unexpectedly higher than that for Enterobacteriaceae. This response could suggest that even these beneficial bacteria can become inflammatory targets in the dysregulated immune environment of UC. Alternatively, this different reactivity could represent a regulatory response that represents an attempt to control these bacterial populations to maintain homeostasis. The lack of a clear difference in the level of IgG reactivity between active and remission stages supports that these IgGs are not simple markers of acute inflammation. Rather, the immune system may remain “sensitized” to specific gut microbes even during remission, persisting as a sort of immunological “ember” that could contribute to future relapse.

Many clinical trials for UC reported that treatment with probiotic *Lactobacillus* spp. and closely related bacterial species reduced gut mucosal inflammation. *Lactobacillus* spp. can induce IL-10-producing M2 macrophages via TLR2/STAT signaling [35]. In addition, IgG reactivity toward normal gut microbiota has been shown to extend to pathogenic bacteria, protecting the host from infections by inhibiting the entry of pathogenic bacteria to the circulation [36]. Therefore, IgGs secreted into the luminal fluids are likely to bind *L. paracasei* or *L. plantarum* to suppress gut mucosal inflammation by facilitating the uptake of these bacteria by macrophages or dendritic cells. Along this line, we examined the cross-reactivity of serum IgG toward *E. coli* and *L. paracasei* or *L. plantarum* (Figure 4 and Figure S2). During remission, absorption of IgG to *L. paracasei* comprehensively reduced subsequent reactivity against *L. paracasei* as well as against all other tested bacteria, including other lactobacilli and Enterobacteriaceae. However, when active-stage serum IgG was absorbed to *L. paracasei*, reactivity to other bacteria decreased, while reactivity to *L. paracasei* itself did not. This result suggests that the serum IgG titer was lower in the active stage than in the remission stage. Meanwhile, after IgG absorption with *E. coli*, no reduction in reactivity to *L. paracasei* or *L. plantarum* was observed (Figure 5). Instead, a trend toward increased reactivity to *L. paracasei* and *L. plantarum* was seen in both active and remission phases. This phenomenon could potentially be explained by a “relative enrichment” effect, wherein the removal of a fraction of anti-*E. coli* IgG may increase the relative population of IgG having other reactivities, such as toward *L. paracasei* and *L. plantarum*, which leads to an enhanced signal.

*E. coli*/IgG immune complexes (IC) strongly activated the classical complement pathway (Figure 6). On the other hand, complement activation by *L. paracasei*/IgG IC was weak, and *L. paracasei* inhibited complement activation by *E. coli*/IgG IC. The complement pathway activation by IC is initiated by the hexa-oligomerization of IgG [37,38]. Cruz et al. reported that Staphylococcal protein A (SpA) hampers this oligomerization and inhibits complement activation, thus evading killing by the complement system [39]. In addition, IgG subclass-3 targeting *S. aureus* elicits complement pathway activation and opsonophagocytosis, even in the presence of SpA [40]. The mode of binding and subclasses of IgG that bind *L. paracasei* should be determined in future studies to understand the role of high serum IgG reactivity to mucosal *L. paracasei* in IBD. However, the suppressive effect of *L. paracasei* on complement activation was not uniform across patients with UC in this study cohort and instead was highly patient-specific. This inter-individual variability may be attributed to differences in IgG subclass profiles (e.g., pro-inflammatory IgG1/IgG3 vs. weakly activating IgG4) or in the specificity of antibodies induced by the patient’s autologous microbiota. This finding suggests that a “one-size-fits-all” strain is not viable for microbial therapies like probiotics or fecal microbial transplantation (FMT).

This study has some limitations, including a small cohort size, and the need to identify bacterial cell surface molecules that react with serum IgG. In addition, this study included only five patients for whom serum and CLFs were collected during both active and remission disease stages, and the disease history (relapse frequency or disease duration) was not considered. A longitudinal study is needed to elucidate the role of the humoral immune response to MAMs for control of gut inflammation in pediatric UC. In addition, metagenomic analysis of gut microbiota is needed to validate our hypothesis that the increase in the population of lactobacilli in MAM and the cross-reactivity of IgG toward other microbes plays a role in suppressing gut mucosal inflammation in pediatric UC. The UC cohort in this study included patients who were temporarily administered antibiotics, corticosteroids, immunomodulators and/or biologics. Patients receiving antibiotics and immunomodulators showed a trend toward lower serum IgG reactivity to gut microbial extracts, whereas administration of corticosteroids and biologics had no effect. We speculate that this is due to a reduction in antigenic stimulation by antibiotics and broad immunosuppression by immunomodulators, which in turn lowered serum IgG reactivity. However, these patients received therapeutics in various combination, making it difficult to draw reliable conclusions due to the cohort size used in this study. Longitudinal studies are crucial for elucidating the impact of therapeutic interventions on serum IgG response to gut microbes.

Many clinical trials have evaluated the effectiveness of *Lactobacillus*-based probiotics on disease activity control for IBD, but the therapeutic effect is still controversial. In addition, IBD is an attractive target for FMT in clinical trials [41]. However, consensus opinion concerning this approach for IBD is not established. Side effects of FMT on IBD patients have also been reported [42,43]. This study demonstrated the importance of using matched samples (serum, MAMs and isolated bacteria) to address the role of humoral immune response to MAMs. Our findings provide useful information to consider treatment with probiotics or FMT that mimics and establishes microbiota similar to self MAMs. Metagenomic analysis of IgG-directed MAMs, identification of the *E. coli* or *Lactobacillus* spp. epitopes that are recognized by serum IgG and the mode of IgG-binding to *L. paracasei* should also be determined in future studies. Together, such findings could help establish effective tailored treatments involving probiotics or FMT.

## 4. Materials and Methods

### 4.1. Sample Collection

Serum (*n* = 33), colonoscopic lavage fluids (CLFs, *n* = 145) and feces were collected from pediatric patients with UC (*n* = 13) and non-IBD patients (*n* = 15) after obtaining informed consent from their guardians (Table 1). For five patients with UC, serum and CLFs were collected at different stages of disease activity (Tables S1 and S2). CLFs were collected from the ascending (A), transverse (T) and sigmoid (S) parts of the colon and rectum (R) during clinical follow-up of the patients at the Pediatrics of Kagawa University Hospital. CLFs were processed and stored at −80 °C immediately after collection. Serum was also stored at −80 °C until use.

### 4.2. Purification of Serum IgG

The IgG fraction of patient serum samples was purified by affinity chromatography using Protein G HP SpinTrap columns (GE Healthcare Japan, Tokyo, Japan), according to the manufacturer’s instructions. Serum IgG purity was evaluated by SDS-PAGE, and the protein concentration was measured with a BCA Protein Assay Kit (Thermo Scientific-JP, Tokyo, Japan).

### 4.3. Protein Extraction from CLFs and Feces

Feces (F) were suspended in 1× phosphate-buffered saline (PBS, pH7.4) to 0.1 g/mL and pelleted by centrifugation at 14,010× *g* for 5 min at 4 °C. CLFs were centrifuged at 14,010× *g* for 5 min at 4 °C to collect bacterial cells. Protein extracts from CLFs and feces were prepared with a MINUTE total protein extraction kit, which allows for protein extraction from thick-walled microbes (cat. no. yt-015, Invent Biotechnology, Inc. Plymouth, MN, USA), according to the manufacturer’s instructions. In brief, the collected samples (A/T/S/R/F) were washed once with 1 mL 1× PBS (pH 7.4) followed by washing with 1 mL distilled water. After resuspending the pellets in 20 μL denaturing buffer, protein extraction powder (80–90 mg) was added, and the suspensions were ground by hand for 2 min. Then, 200 μL denaturing buffer was added and the suspensions were ground for another 30 s to maximize protein extraction. Finally, the suspensions were centrifuged at 15,000× *g* for 1 min at 4 °C, and the supernatants were used as the protein extracts. The total protein concentration was measured using a Pierce BCA Protein Assay Kit (Thermo Scientific-JP, Tokyo, Japan). Extracts were stored at −80 °C until use.

### 4.4. Isolation of Gut Microbes from Feces

Fecal microbes from stored stock cultures or that were newly-isolated from pediatric patients with UC were used (Table S3). Isolates from patients were obtained using McConkey’s agar (for Enterobacteriaceae), EF agar (for Enterococcaceae) or MRS agar plates (for Lactobacillaceae). The feces samples were suspended in 1× PBS (pH7.4) to 0.1 g/mL, and 10-fold serial dilutions (100 μL) made with 1× PBS (pH7.4) and plated onto these agar plates. McConkey agar and EF agar were cultured aerobically, whereas MRS agar was cultured anaerobically using AnaeroPacks (Mitsubishi Gas Chemical Co. Inc., Tokyo, Japan). Identification of bacterial species in the fecal isolates was carried out using 16S rRNA gene sequencing. In brief, PCR was performed on colonies using 27F (5′-AGAGTTTGATCMTGGCTCAG-3′) and 1492R (5′-CGGTTACCTTGTTACGACTT-3′) primers. The amplified products were sequenced and the obtained sequences were used in homology searches of the NCBI BLAST database (https://blast.ncbi.nlm.nih.gov/Blast.cgi, accessed on 1 July 2025)

### 4.5. Protein Extraction from Fecal Isolates from Pediatric Patients with UC

Each fecal isolate was anaerobically cultured in 10 mL Brain Heart Infusion broth (BHIS; Eiken Chemical Co., Ltd., Tokyo, Japan) supplemented with 0.5% yeast extract (Becton Dickinson and Company, Tokyo, Japan), 0.1% L-cysteine hydrochloride monohydrate (Merk KGaA, Darmstadt, Germany), 0.375% D-glucose (Nacalai Tesque Inc. Kyoto, Japan) and 5 µg/mL hemin (Merk KGaA, Darmstadt, Germany) for 24 h at 37 °C. After overnight incubation, 5 mL of the bacterial culture was centrifuged at 15,000× *g* for 5 min at 4 °C, and the pellets were resuspended in 1× PBS (pH 7.4) containing complete protease inhibitor cocktail (Roche Diagnostics K.K. Tokyo Japan). The suspensions were then sonicated for 5 min with 1 min intervals in an ice bath. After sonication, the samples were centrifuged at 10,000× *g* for 10 min at 4 °C, and the supernatants were used as protein extracts derived from the isolates. Protein extracts were stored at −20 °C until use.

### 4.6. Serum IgG Reactivity to Protein Extracts from CLFs, Stools or Fecal Isolates

Western slot blots were performed to compare the reactivity of serum IgG to that for extracts from CLFs, stools or fecal isolates from pediatric patients with UC and from non-IBD patients. Protein extracts (80 μg) from CLFs or fecal isolates were blotted onto nitrocellulose membranes (0.45 µm, Bio-Rad Laboratories Inc., Feldkirchen, Germany) using a Bio-Dot SF microfiltration device (Bio-Rad Laboratories Inc., Hercules, CA, USA), according to the manufacturer’s instructions. Human IgG (Fujifilm Wako Pharmaceuticals, Osaka, Japan) was used as a standard to calculate the relative reactivity of serum IgG to the extracted proteins. After drying for 1 h, the nitrocellulose membranes were washed three times with 1× Tris-buffered saline (TBS), and then blocked with 3% skim milk overnight. The membranes were washed three times with 50 mL 1× TBS and probed with 3 μL of the purified IgG from the corresponding patient’s serum (1:100 dilution) for 1 h in a hybridization incubator (HB-80, TAITEC Co. Tokyo, Japan). Then, the membranes were washed three times with 50 mL 1× TBS and incubated with 2 μL goat anti-human IgG (H+L)-HRP (A18811, Thermo Scientific-JP, Tokyo, Japan, 1:1000 dilution) for 1 h. After washing three times with 1× TBS, 1 mL of chemiluminescence reagent, Amersham^TM^ ECL^TM^ Prime Western Blotting Detection Reagent (Cytiva, Tokyo, Japan) was used to detect IgG binding. Slot blot images were captured using an ImageQuant LAS-4000 system (GE Healthcare Japan, Tokyo, Japan). The chemiluminescence exposure time was set to 1 min for all images. Band intensities were analyzed using ImageJ Software (ImageJ for Windows, version 1.8.0, Softonic International, Barcelona, Spain). The relative amount of bound IgG was calculated based on the standard curve obtained from band intensities for the IgG standard (3.05 to 48.82 ng/mL).

### 4.7. Preparation of L. paracasei-, L. plantarum- or E. coli-Absorbed Serum IgG

*L. paracasei* OY7, *L. plantarum* OY1 and *E. coli* TF10, which were previously isolated from pediatric patients with active UC (Table S2), were cultured anaerobically in BHIS for 24 h at 37 °C. Then, 1 mL of the bacterial cultures was washed with 1 mL 1× PBS (pH7.4) and the cell densities were measured at 600 nm and adjusted to OD_600_ = 1.0. The bacterial cell suspensions (1 mL of OD_600_ = 1.0) were centrifuged at 15,000× *g* for 5 min at 4 °C, and the pellets were suspended in 1 mL 4% paraformaldehyde (PFA) and incubated at room temperature for 30 min. The PFA-fixed cells were collected by centrifugation at 3500× *g* for 15 min at 4 °C and washed three times with an equal volume of 1× PBS (pH7.4). The purified serum IgG (S25 or S36) collected from pediatric patients with UC (UCR25 or UCA36) was mixed with the PFA-fixed bacterial cell suspension (1:100) and incubated for overnight at 4 °C with rotation. Finally, the mixtures were centrifuged at 15,000× *g* for 5 min at 4 °C, and the supernatants were collected. The supernatants were stored at −20 °C until use. Western slot blot analysis was performed as described above to determine whether serum IgG reactivity to the fecal isolates was altered after absorption by *L. paracasei*, *L. plantarum* or *E. coli*.

### 4.8. Complement Pathway Activation by Immune Complexes in IgG-Fecal Isolates

Activation of the complement pathway by immune complexes comprising serum IgG and *E. coli* or *L. paracasei* was examined using a Wieslab Complement System Classical Pathway Kit (Svar Life Sciences, Malmö, Sweden), according to the manufacturer’s instructions. This kit is an ELISA-based tool to detect the C5-C8 membrane attack complex (MAC). We used *E. coli* and *L. paracasei* isolated from both patients who had active UC or from patients whose UC was in remission. Two pairs of strains were used in this assay: *E. coli* KK19 and *L. paracasei* KT1 were isolated from UCR8 (remission) and UCA9 (active), respectively (PAIR-1 in Table S1). *E. coli* KT20 and *L. paracasei* KK2 were isolated from UCR25 (remission) and UCA26 (active), respectively (PAIR-3 in Table S1). All isolates were anaerobically cultured in BHIS broth overnight at 37 °C. After adjusting the OD_600_ of the culture to 1.0 with 1× PBS (pH7.4), the bacterial cell suspensions were heat-inactivated at 60 °C for 1 h. Then, 2 μL heat-inactivated microbial cells (*E. coli* and/or *L. paracasei*) were mixed with 20 μL of serum from the matched patient. As a negative control, 2 μL 1× PBS (pH7.4) was added to the serum in place of the microbial cell suspension. The samples were incubated at 37 °C for 30 min, before MAC formation was stopped by the addition of 0.5 M EDTA (final concentration, 10 mM). The samples were then centrifuged at 3220× *g* for 15 min at 4 °C. MAC formed in the reaction mixture was quantified by ELISA using a antibody specific to MAC.

### 4.9. Statistical Analysis

All statistical analyses were performed using R software (v.4.4.2). A *p*-value of <0.05 was considered statistically significant unless otherwise noted.

#### 4.9.1. Generalized Linear Mixed-Effects Models (GLMMs)

Primary statistical analyses in this study were conducted using generalized linear mixed-effects models (GLMMs) with the glmmTMB package (v.1.1.11). GLMMs are a statistical method that can simultaneously account for random effects, such as inter-individual differences and non-normal data distributions. To account for the data dependency from repeated measurements of serum samples collected from each patient, a nested random effect was specified in all models. To appropriately model the distribution of the outcome variable, serum IgG reactivity, a Gamma or Tweedie distribution with a log link function was specified. The Tweedie distribution was specifically applied when the data included zero values. Fixed effects in the models included disease activity, clinical conditions, PUCAI score, Matts score, microbe type, and their interactions. Following model construction, post hoc pairwise comparisons were performed using the emmeans package (v.1.11.1), with *p*-values adjusted using the Tukey method.

#### 4.9.2. Other Comparisons

Clinical parameters between the non-IBD and UC groups were compared using the Mann–Whitney U test, excepting that differences in the proportions of categorical variables, such as gender, antibiotic administration, and fecal occult blood (FOB) status, were evaluated between the non-IBD and UC groups using Fisher’s exact test (Table 1). For site-specific comparisons between the active and remission stage of UC, paired *t*-tests were used (Figure 1C). For the absorption assays, comparisons of IgG reactivity before and after absorption were performed using two-sample *t*-tests (Figure 4, Figure 5 and Figure S2). For the complement activation assay, comparisons among the different conditions within each serum sample were performed using a one-way analysis of variance (ANOVA) followed by Tukey’s post hoc test (Figure 6).

## Figures and Tables

**Table 1:** Characteristics of enrolled patients.

	UC	Non-IBD	*p*-Value ***
Number of patients	13	15	-
%Male	46	60	0.729 ^(a)^
Age	5 to 17	4 to 16	0.139 ^(a)^
%Antibiotics	30.8	6.67	0.346 ^(a)^
Number of serum samples	18	15	-
Disease activity			
Active	9	-	-
Remission	9	-	-
Numbers of samples (total)	83	62	-
Ascending colon	17	15	-
Transverse colon	17	11	-
Sigmoid colon	18	14	-
Rectum	17	13	-
Feces	14	9	-
Matts grade			
1	23	-	-
2	29	-	-
3	17	-	-
Blood analysis			
White blood cell *	8989 ± 4093	6889 ± 4220	0.0247
Hemoglobin *	12.1 ± 1.61	13.8 ± 1.24	0.0014
Hematocrit *	36.5 ± 4.56	39.3 ± 3.29	0.0858
Platelet *	32.3 ±10.4	25.4 ± 5.20	0.0399
ESR *	17.0 ± 15.3	7.54 ± 5.05	0.123
Serological analysis			
Albumin *	3.97 ± 0.777	4.51 ± 0.352	0.0236
C-reactive protein **	0.04 (0.01–4.6)	0.01 (0.01–0.19)	0.11
Serum amyloid A **	14.8 (0.3–354)	1.6 (0.2–6.3)	0.0971
IgG *	1330 ± 364.5	1050 ± 237.8	0.0222
IgM *	125 ± 74.2	107 ± 81.6	0.336
IgA *	166 ± 44.3	141 ± 50.6	0.232
%FOB	60	16.7	0.0473 ^(a)^
Fecal calprotectin **	1515 (47.3–14,400)	33.4 (7.6–456)	0.0002

Abbreviations: FOB: fecal occult blood, IBD: inflammatory bowel disease, UC: Ulcerative colitis. * mean ± SD (standard deviations), ** median, IQR (inter quantile range). *** The Mann–Whitney U test was used to compare the difference between the Non-IBD and UC groups. A *p*-value < 0.05 was considered statistically significant. ^(a)^ The association between patient groups (Non-IBD vs. UC) was analyzed using Fisher’s exact test.

**Table 2:** Summary of GLMM evaluating the interaction of serum IgG reactivity with UC disease activity and clinical trend.

Predictor	Estimate (β) ^4^	Std. Error (SE)	95% CI	Ratio (exp(β)) ^3^	*p*-Value
(Intercept) ^1^	2.07	0.42	[1.24, 2.90]	—	<0.001
Disease activity: Remission	−1.77	0.55	[−2.84, −0.69]	0.17	0.001
Clinical Trend: Improve	−3.04	0.55	[−4.12, −1.95]	0.05	<0.001
Interaction Term ^2^	4.36	0.78	[2.83, 5.88]	77.9	<0.001

^1^ (Intercept): Predicted value on the log scale for the reference group (Active phase and Exacerbated trend). ^2^ Interaction Term: Disease activity: Remission x Clinical Trend: Improved. ^3^ Ratio (exp (β)): Exponentiated coefficient, representing the multiplicative change in the mean serum IgG reactivity compared to the reference group. ^4^ Estimate (β): Effect size on a logarithmic (log) scale.

## Data Availability

Data used to generate the figures and tables can be provided upon reasonable request to the corresponding author.

## Supplementary Materials

The following supporting information can be downloaded at https://www.mdpi.com/article/10.3390/ijms26178196/s1.

## Abbreviations

The following abbreviations are used in this manuscript:

UC	Ulcerative colitis
IBD	Inflammatory bowel disease
LM	Luminal microbes
MAM	Mucosa-associated microbes
IBDS	Inflammatory bowel diseases
OTUs	Operational taxonomic units
sIgA	Secretory IgA
FcRn	Neonatal Fc receptor
Ag	Antigen
CLFs	Colonoscopic lavage fluids
A	Ascending colon
T	Transverse colon
D	Descending colon
S	Sigmoid colon
R	Rectum
F	Feces
PUCAI	Pediatric UC activity index
Std	Standard
PFA	4% Paraformaldehyde
PVDF	Polyvinylidene difluoride
UC-A	Ulcerative colitis active
UC-R	Ulcerative colitis remission
LSA	*Ligilactobacillus salivarius*
LPL	*Lactiplantibacillus plantarum*
LPC	*Lactocaseibacilus paracasei*
Eco	*Escherichia coli*
Kpn	*Klebsiella pneumoniae*
Pmi	*Proteus mirabilis*
Efa	*Enterococcus faecalis*
LPC-UCA	*L. paracasei*-absorbed Ulcerative colitis active serum
LPC-UCR	*L. paracasei*-absorbed Ulcerative colitis remission serum
LPL-UCA	*L*. *plantarum*-absorbed Ulcerative colitis active serum
LPL-UCR	*L*. *plantarum*-absorbed Ulcerative colitis remission serum
EC-UCA	*E. coli*-absorbed Ulcerative colitis active serum
EC-UCR	*E. coli*-absorbed Ulcerative colitis active serum
BHIS	Brain Heart Infusion Agar
PBS	Phosphate Buffered Saline
