# Supplementary figures and images for: Reactivity of Autologous Serum IgG to Gut Microbes in Pediatric Ulcerative Colitis
Source: Int J Mol Sci. 2025 Aug 23;26(17):8196. doi: 10.3390/ijms26178196 (PMC12428623; doi:10.3390/ijms26178196)

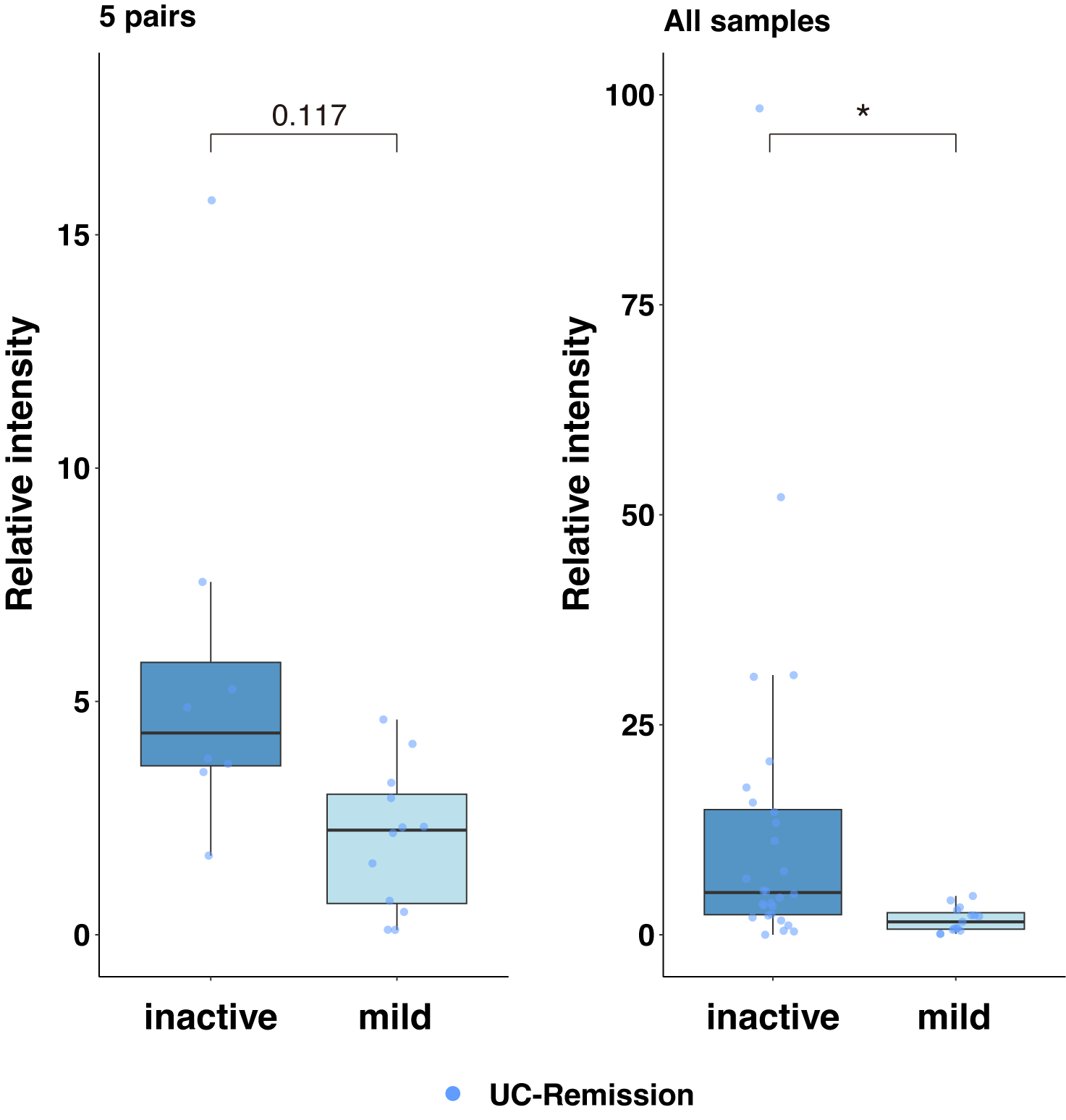

Supplement: Supplementary file 1 [file ijms-26-08196-s001.zip › Figure S1.png]

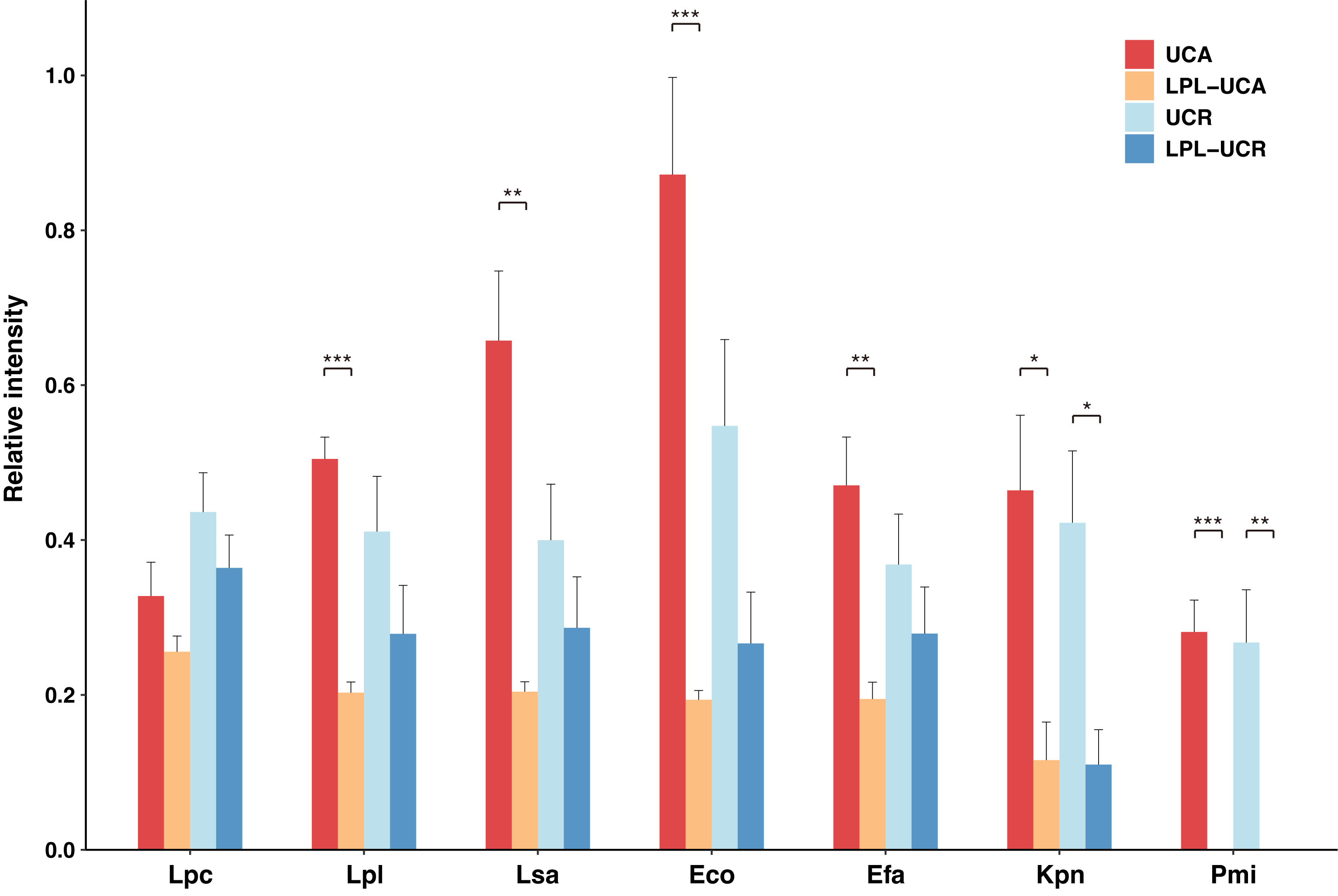

Supplement: Supplementary file 1 [file ijms-26-08196-s001.zip › Figure S2.png]
